# Polysemia as a concept to understand the encoding of sensory information

**DOI:** 10.3389/fnins.2025.1588437

**Published:** 2025-05-14

**Authors:** Christian Ethier, Zohreh Vaziri, Martin Deschênes

**Affiliations:** CERVO Brain Research Center, Université Laval, Quebec, QC, Canada

**Keywords:** Polysemia, sensory processing, trigeminal system, inhibitory control, context-dependent perception, vibrissal pathway

## Abstract

In this article, we explore the concept of polysemia in sensory information processing within the brain. We suggest that, just as words can have different meanings based on context, sensory inputs are interpreted differently depending on the animal’s current state and behavior. Focusing on the trigeminal sensory nuclei in rats, we highlight the role of inhibitory circuits in gating sensory information and propose that sensory signals are polysemic, with their meaning influenced by emotional, hormonal, and motivational factors.

## Introduction

The broad concept of polysemia refers to the co-existence of multiple related meanings in a single form. It is commonly discussed in the context of language, referring to words or phrases that have multiple related meanings. In languages, where this concept is mainly seen, polysemia refers to the notion that the meaning of a word written the same way and pronounced the same way is context dependent. For example, consider the word “head,” which has various meanings in many languages such as English, French, and Farsi. Its literal meaning refers to the top part of the human or animal body, but its polysemous extensions may refer to a leader (head of the department), the start position (head of the page) and the tip or top of something (mountain head, nail head). While the word is the same, its meaning is usually made clear from the context in which it is used.

## Polysemia in sensory processing

Here, we explore whether the concept of polysemia can help us better understand how sensory information is encoded in the brain. Just as the meaning of a word can shift depending on context, we propose that a given pattern of neuronal activity in sensory pathways can be interpreted differently by the nervous system depending on the animal’s internal state and external circumstances. Specifically, we argue that the behavioral outcome of a sensory message depends on behavioral, emotional, hormonal, and motivational states, as well as on prior experiences. This hypothesis is grounded in accumulating evidence that sensory processing is not a fixed relay of information but is dynamically modulated by context-dependent factors even at early stages of the sensory pathways (Ferezou et al., 2007).

Let us consider the deflection of a mystacial vibrissa in rats. It may result from the animal self-movement, an air current, the interaction with objects or other members in a colony. Thus, a train of action potentials in primary vibrissa afferents is polysemic, in the sense that it has different meanings depending on the state and current behavior of the animal. One of the central issues in sensory physiology is to understand how an animal endowed with highly sensitive sensory organs can control the unceasing stream of sensory inputs it receives and select those that are most relevant to an adaptive behavior. When rats whisk to explore a new environment, they are likely little interested in object texture, no more than we are when we stretch out our arms to locate obstacles in a dark room. Processing texture information in this context appears behaviorally irrelevant, and there is no good reason to believe that this information should be conveyed to the cerebral cortex through the lemniscal pathway.

As proposed by Bendixen et al. (2012), perception is not a passive process of registering sensory signals; instead, it is an active interpretation of the information that impinges on our senses. A key factor in this process is that the brain automatically processes sensory inputs in a context-relevant manner.

## Neural mechanisms of sensory polysemia

**Figure 1:**
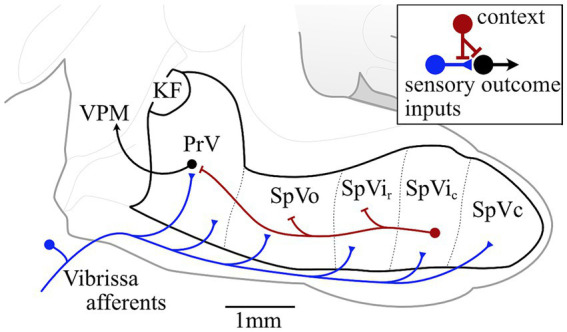
Circuit diagram of intersubnuclear projections of GABAergic interpolaris neurons that gate the relay of vibrissa information in rats principal trigeminal nucleus. Abbreviations: KF, Kölliker-Fuse nucleus; PrV, principal sensory trigeminal nucleus; SpVo, spinal trigeminal nucleus pars oralis; SpVir, rostral part of the spinal trigeminal nucleus pars interpolaris; SpVic, caudal part of the spinal trigeminal nucleus pars interpolaris; SpVc, spinal trigeminal nucleus pars caudalis; VPM, ventral posteromedial nucleus of the thalamus.

We propose that sensory signals associated with different modes of tactual information processing are polysemic and differentially gated in brainstem trigeminal nuclei by inhibitory intersubnuclear projections. The trigeminal sensory nuclei are the first stations of sensory processing in the whisker-to-barrel pathway (Figure 1). In addition to projection neurons that ascend the neuraxis in distinct sensory pathways (see review by Deschênes and Urbain, 2009), trigeminal sensory nuclei are replete with pre-and postsynaptic inhibitory connections (Bae et al., 2000; Avendaño et al., 2005; Furuta et al., 2008) which gate the relay of sensory messages. If it were not of tonic inhibition the animal would be hyper-reactive to any stimulus. The high proportion of Gamma-Aminobutyric Acid (GABA) interpolaris neurons that project to the principal trigeminal nucleus (PrV) (86%) (Furuta et al., 2008) provides compelling evidence for a strong inhibitory control of synaptic transmission in the lemniscal pathway, carrying mechanosensation from the PrV to the thalamic VPM nucleus and higher centers.

Trigeminal sensory nuclei are replete with axo-axonic endings (review by Bae and Yoshida, 2011). In the spinal cord the inhibitory interneurons that form axo-axonic contacts with sensory afferents, responsible for presynaptic inhibition, differ from other GABAergic neurons in that they alone express Glutamic Acid Decarboxylase-65 (GAD65), one of two GABA-synthetic enzymes (Hughes et al., 2005). GABAergic cells expressing GAD65 are present in the caudal sector of the interpolaris nucleus (SpVic), which projects to the trigeminal sensory nuclei (Guthmann et al., 1998). This suggests that GABAergic SpVic cells may gate sensory transmission in the lemniscal pathway via presynaptic or postsynaptic inhibition.

## Discussion

The concept of sensory polysemia illustrates the dynamic and context-dependent nature of sensory information processing. Traditional models of sensory transmission emphasize hierarchical pathways that faithfully relay external stimuli to higher-order structures. However, recent findings suggest that sensory signals are actively shaped by the animal’s internal state and behavioral goals before reaching cortical areas (Furuta et al., 2008).

The extensive inhibitory gating within the trigeminal sensory nuclei provides a compelling mechanism for this selectivity. By differentially modulating sensory relay based on emotional, hormonal, and motivational factors, these circuits ensure that only behaviorally relevant information is prioritized for further processing. This aligns with broader perspectives in sensory physiology, where perception is increasingly recognized as an interpretative process rather than a mere passive registration of stimuli.

## Conclusion

Just as polysemia in language reflects the flexible interpretation of words based on context, sensory processing in the brain relies on dynamic gating mechanisms to extract meaning from incoming stimuli. By integrating internal states with external inputs, the nervous system ensures that perception remains adaptive and behaviorally relevant, reinforcing the idea that sensory signals do not carry fixed meanings but are actively shaped by the organism’s needs. Our proposal that sensory signals are polysemic and differentially gated in the brainstem introduces a novel framework for understanding early sensory processing as inherently interpretative and context-dependent. Although our discussion draws from animal studies, the principle of sensory polysemia likely extends to humans, providing a broader conceptual basis for understanding the general mechanisms of sensory processing. This perspective challenges traditional feedforward models and opens new avenues for investigating how internal states influence perception at the earliest stages of the sensory hierarchy.

## Data Availability

The original contributions presented in the study are included in the article/supplementary material, further inquiries can be directed to the corresponding author.
